# Impact of incorporating visual biofeedback in 4D MRI

**DOI:** 10.1120/jacmp.v17i3.6017

**Published:** 2016-05-08

**Authors:** David T. To, Joshua P. Kim, Ryan G. Price, Indrin J. Chetty, Carri K. Glide‐Hurst

**Affiliations:** ^1^ Department of Radiation Oncology Henry Ford Health System Detroit MI USA

**Keywords:** MR‐simulation, 4D MRI, visual biofeedback

## Abstract

Precise radiation therapy (RT) for abdominal lesions is complicated by respiratory motion and suboptimal soft tissue contrast in 4D CT. 4D MRI offers improved contrast although long scan times and irregular breathing patterns can be limiting. To address this, visual biofeedback (VBF) was introduced into 4D MRI. Ten volunteers were consented to an IRB‐approved protocol. Prospective respiratory‐triggered, T2‐weighted, coronal 4D MRIs were acquired on an open 1.0T MR‐SIM. VBF was integrated using an MR‐compatible interactive breath‐hold control system. Subjects visually monitored their breathing patterns to stay within predetermined tolerances. 4D MRIs were acquired with and without VBF for 2‐ and 8‐phase acquisitions. Normalized respiratory waveforms were evaluated for scan time, duty cycle (programmed/acquisition time), breathing period, and breathing regularity (end‐inhale coefficient of variation, EI‐COV). Three reviewers performed image quality assessment to compare artifacts with and without VBF. Respiration‐induced liver motion was calculated via centroid difference analysis of end‐exhale (EE) and EI liver contours. Incorporating VBF reduced 2‐phase acquisition time (4.7±1.0 and 5.4±1.5min with and without VBF, respectively) while reducing EI‐COV by 43.8%±16.6%. For 8‐phase acquisitions, VBF reduced acquisition time by 1.9±1.6min and EI‐COVs by 38.8%±25.7% despite breathing rate remaining similar (11.1±3.8 breaths/min with vs. 10.5±2.9 without). Using VBF yielded higher duty cycles than unguided free breathing (34.4%±5.8% vs. 28.1%±6.6%, respectively). Image grading showed that out of 40 paired evaluations, 20 cases had equivalent and 17 had improved image quality scores with VBF, particularly for mid‐exhale and EI. Increased liver excursion was observed with VBF, where superior–inferior, anterior–posterior, and left–right EE‐EI displacements were 14.1±5.8, 4.9±2.1, and 1.5±1.0 mm, respectively, with VBF compared to 11.9±4.5, 3.7±2.1, and 1.2±1.4 mm without. Incorporating VBF into 4D MRI substantially reduced acquisition time, breathing irregularity, and image artifacts. However, differences in excursion were observed, thus implementation will be required throughout the RT workflow.

PACS number(s): 87.55.‐x, 87.61.‐c, 87.19.xj

## I. INTRODUCTION

Four‐dimensional CT (4D CT) is currently the standard of care for providing the respiratory‐induced excursion information needed for treatment planning in the abdominal region.^1^, ^2^ 4D CT can provide the necessary data for a variety of radiation treatment planning techniques: motion‐encompassing, respiratory gating, breath‐hold, forced shallow‐breathing, and respiration‐synchronized techniques.^(3)^ However, unlike lung lesions, liver, pancreatic, and other abdominal lesions are embedded in soft tissue, making it difficult to distinguish tumor boundaries from healthy tissue. Magnetic resonance imaging (MRI) provides improved soft tissue contrast, eliminates imaging radiation dose, and allows flexibility for imaging in different orientations.^4^, ^5^ Clinically available MRI motion management techniques rely on either acquiring images at breath‐hold or triggering acquisition at the end‐exhale (EE) breathing phase as determined by internal or external surrogates.^6^, ^7^, ^8^ As a result, facilities that use midventilation^(9)^ or internal target volume (ITV)^(10)^ treatment approaches will be limited. While fast cine‐MRI has been used to provide respiratory information,^6^, ^11^, ^12^ this method only provides information in two dimensions, which may inadequately characterize out‐of‐plane abdominal lesion motion. Recently, two interleaved orthogonal cine‐MRI scan planes were shown to elucidate 3D organ trajectories, although this approach also increases the time lapse between two scans in the same plane.^(13)^ To delineate lesions and characterize excursion for radiation therapy purposes, three dimensions are necessary.

Four‐dimensional magnetic resonance imaging (4D MRI) provides an alternative strategy that more readily compares to 4D CT. Recently, Hu et al.^(14)^ developed a novel prospective, amplitude‐based triggering technique to acquire T2‐weighted single‐shot turbo spin echo (TSE) images at defined phases of the respiratory cycle. One of the major advantages of Hu's technique is that acquiring different respiratory phases consecutively during a single respiratory cycle would no longer be necessary. However, because this 4D MRI algorithm is prospectively triggered based on amplitude, long acquisition times were reported due to recurring pauses arising from highly irregular breathing patterns.^14^, ^15^, ^16^ It has been shown that incorporating audio coaching and visual biofeedback (VBF) into 4D CT acquisition and radiation therapy delivery improves breathing regularity, increases anatomic reproducibility, and reduces the overall time burden of these procedures.^17^, ^18^, ^19^ Given long 4D MRI exam times, efforts to reduce breathing irregularities are advantageous. This study evaluates the impact of using VBF during 4D MRI acquisition, with the overarching goal of improving scan efficiency and patient breathing regularity to support an MR‐only workflow.

## II. MATERIALS AND METHODS

### A. Volunteer study

Ten healthy volunteers (average age: 29.8±9.9 yr [22–56 yr]) were consented to a prospective, IRB‐approved imaging protocol. All data were acquired with the subjects in the head‐first supine position with arms positioned above their heads to allow for body coil placement. Subjects were scanned using a large, rigid eight‐element phased array coil. Both the bellows (used for VBF) and air‐filled cushion (for 4D MRI triggering) were placed on the subjects' abdomens in the region of maximum anterior–posterior excursion, typically occurring between the xiphoid process and the umbilicus. Eight‐phase 4D MRI acquisitions were performed with and without VBF for all subjects. Eight phases were selected based on a previous 4D MRI optimization study conducted by our group.^(16)^ For comparison, 2‐phase acquisitions were acquired for seven of the volunteers.

### B. 4D MRI algorithm and acquisition

The 4D MRI algorithm used in this study consists of prospective respiratory amplitude‐triggering of a 2D multislice single‐shot, T2‐weighted TSE sequence.^(14)^ Slices are acquired in an interleaved order, as described in Hu et al.^(14)^ Images are only acquired when the respiratory signal reaches a predetermined trigger level, each acquired image will correspond to a specific respiratory state, and each slice is acquired at each respiratory state only once yielding ∼40−50 images per phase. Images were acquired in the coronal plane with the following parameters: $\text{TE}/\text{TR}/\text{flip angle}=82/3831–4876\;\text{ms}/90^{\circ }$, pixel bandwidth=259Hz/pixel, in‐plane spatial resolution $3\times 3\;{\text{mm}}^{2}$, voxel size $\approx 0.98\times 0.98\times 4-5\;{\text{mm}}^{3}$, and $\text{FOV}=450\times 450\times 210-246\;{\text{mm}}^{3}$. To improve scan efficiency, sensitivity encoding was enabled for accelerated acquisition (SENSE=1.7). Coronal acquisitions are performed to ensure high spatial resolution in the superior–inferior direction where liver motion is dominant.^(3)^ 4D MRIs were acquired on a 1.0T Philips Panorama High Field Open (HFO) magnetic resonance system (RT Oncology Configuration, v3.5.2, Philips Medical Systems, Cleveland, OH), which has been described in detail elsewhere.^16^, ^20^ Respiratory triggering was performed based on a signal derived from an air‐filled cushion placed on the abdomen that was used as an external surrogate. The algorithm requires a calibration period of a few breathing cycles to determine the threshold amplitudes at which all phases will be triggered. Each triggering point correlates to a 2D slice of the volumetric image for the corresponding respiratory phase. To improve the efficiency of the initial 4D MRI algorithm, the vendor has implemented additional proprietary improvements to reduce scan time by adapting to changes in breathing pattern.^(16)^ A standalone executable program (Python, version 2.7.3) developed by the vendor, was used for offline analysis and review of respiratory waveforms.

### C. Visual biofeedback

**Figure 1:**
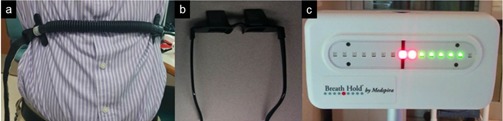
(a) Bellows device on abdomen, (b) rear‐viewing glasses worn by subjects, (c) light‐emitting diode (LED) display calibrated at end‐exhale (red LED at center hash mark) and breathing extent shown.

VBF was integrated using an MR‐compatible interactive breath‐hold control system (Breath Hold ES, Medspira, LLC, Minneapolis, MN). Figure 1 highlights the major components of the system: a bellows pneumatic belt placed on the subject's abdomen, rear‐viewing glasses, and a wireless light emitting diode (LED) display. The green and red LEDs illuminate as the bellows expand and contract with respiration. An initial coaching session was held to allow for breathing assessment and determine an amplitude range consistent with the subject's normal unguided free‐breathing (UFB). To ensure ample detection, patient‐specific sensitivity adjustments were performed based on the bellows response. During an EE breath‐hold, the LED display was calibrated at the center diode (Fig. 1(c)). After the subjects were comfortable with the system, they were instructed to breathe regularly, visually monitor the LED display using rear‐viewing glasses, and stay within predetermined tolerances. Multiple remote LED displays were wirelessly paired with the base unit to enable patient monitoring from the control room.

### D. Scan efficiency and regularity metrics

To quantify differences in scan efficiency with VBF, acquisition time and duty cycle (programmed time/acquisition time) were calculated. Breathing waveform analysis was conducted based on waveforms derived from the air‐filled cushion used for 4D MRI triggering and exported via vendor‐provided research software. Because the baseline amplitude is arbitrary, the minimum amplitude was set to zero arbitrary units (AU) and waveforms were normalized to the maximum EI amplitude. Before a peak‐picking algorithm was used to detect EI peak values, a commonly used least‐square smoothing (i.e., Savitzky‐Golay filter^(21)^) was applied to raw data to reduce noise in the waveforms using commercially available software (OriginLab, Northampton, MA). Using a least‐square filter is advantageous to other smoothing filters, such as adjacent averaging, because it preserves peak heights and widths, thereby preserving the EI amplitude values. Applying the filter to the data ensured that peak picking was not detecting peaks in the local minima of the waveforms. Automated peak‐picking was then performed in OriginLab.

To characterize breathing regularity, the normalized end‐inhale coefficient of variation (EI‐COV), defined as the ratio between standard deviation and mean of the waveform amplitude, was calculated. The breathing rate was defined as the number of EI peaks per minute. Results from scan acquisitions both with and without VBF were compared for all metrics via percent difference: $\frac{\text{UFB}-\text{VBF}}{\text{UFB}}\times 100\%$.

### E. Qualitative artifact review

**Figure 2:**
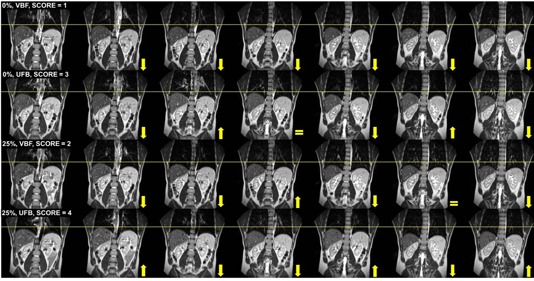
Example of qualitative image consensus grading for Subject 1. Contiguous coronal images are shown from the anterior (left‐most image) to the posterior (right‐most image) with the arrows indicating the primary liver direction from the previous image. Top row shows the end‐inhale 0% phase with a score of 1 using visual biofeedback (VBF) with the liver moving inferiorly for consecutive slices. End‐inhale phase (2nd row) with unguided free‐breathing (UFB) is shown with a score of 3 due to the variable liver positioning on 3 out of 6 consecutive slices. Mid‐end‐exhale (25%) phase (3rd row) with VBF yielding a score of 2 due to discontinuities in liver position on 2 out of 6 consecutive slices. Mid‐end‐exhale (25%) (4th row) with UFB had a score of 4 due to the variable discontinuities and large displacement of the liver on consecutive slices. This case also highlights the image quality improvements obtained when VBF was implemented. VBF=with visual biofeedback, UFB=unguided free breathing,image grading scores defined in text, image grading scores defined in text.

Subjective image quality grading was performed by three experienced readers that formed a consensus group. Coronal displays of the 4D MRI scans with and without VBF were reviewed simultaneously for grading using clinical software (Advanced Viewing workspace, HFO 3.5.2). The EI, mid‐EI (75%), EE, and mid‐EE (25%) were evaluated based on the continuity of the liver dome, yielding 80 phase evaluations overall (40 VBF, 40 UFB). Individual phase datasets were scrolled through and the continuity of the liver dome was evaluated on contiguous slices. Discontinuities in the anatomy were graded according to their severity using the following scale:^(22)^ (1) none to very few artifacts observed (mostly continuous anatomy); (2) minor artifacts not impacting use; (3) major artifacts that impede use; and (4) significant artifacts prohibiting use. Figure 2 shows examples of qualitative consensus grading scores for Subject 1. To avoid reader bias, images were uploaded to the scanner software in a blinded fashion by an MRI technologist who did not participate in image grading. Before consensus grading was performed, two 4D MRI cases not included in the cohort were reviewed to serve as a baseline and establish the grading scale. Grading was compared with and without VBF and results were compared using paired samples *t*‐tests (0.05 level).

### F. Liver excursion

**Figure 3:**
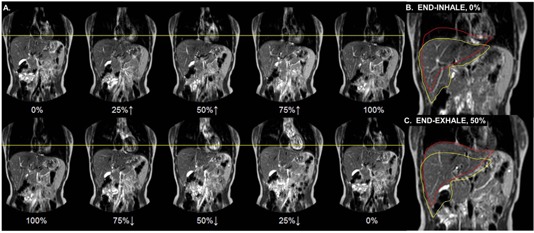
(a) Coronal slice at eight respiratory phases for Subject 4 where 0% indicates end‐exhale (EE) and 100% is end‐inhale (EI). Yellow line=superior dome of liver at EE. ↑=inhalation↓=exhalation. (b) Coronal EI image with the red contour indicating the EE liver position and the yellow contour representing the liver at EI. (c) Corresponding EE image for the same subject. Whole liver excursion was performed via centroid difference calculations between the EI and EE breathing phases.

First, liver contours were manually delineated on the EE phase of the 4D MRI. To facilitate more efficient contouring, an extended multipass B‐spline deformable image registration (DIR) performed in Velocity AI (Velocity Medical Solutions, Atlanta, GA) was used to propagate liver contours from the EE to the EI dataset using a clinically available scripted workflow. Propagated EI liver contours were visually inspected for agreement with the underlying anatomy and then manually adjusted. While DIR was used to propagate contours to improve efficiency, the manual changes made to the propagated contours rendered the underlying displacement vector fields as inaccurate. First, the EE and EI centroid positions of the whole‐liver contours were found. Then, respiratory‐induced excursion was calculated based on the difference between the EE and EI centroids along three orthogonal directions (superior–inferior [S–I], anterior–posterior [A–P], left‐right [L–R]). Figure 3(a) illustrates eight respiratory 4D MRI phases, while Figs. 3(b) and 3(c) highlight the coronal EI and EE liver positions, respectively. This process was conducted for both UFB and VBF datasets and EI‐EE liver centroid differences compared.

## III. RESULTS

### A. Scan efficiency

**Figure 4:**
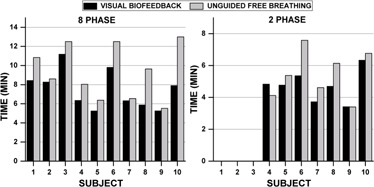
Comparison of 4D MRI scan times with and without visual biofeedback for 8‐ and 2‐phase acquisitions.

Figure 4 shows the scan times both with and without the use of VBF for each subject during 8‐ and 2‐phase acquisitions. For 8‐phase acquisitions, the average scan time reduction was 18.4%±13.2% with VBF (7.5±2.0min vs. 9.4±2.8min without). However, for 2‐phase acquisitions, only a modest reduction (10.3%±15.8%) in average scan time was observed (4.7±1.0min with VBF, 5.4±1.5min without). Subject 10 yielded the longest scan time (13 min) without VBF for an 8‐phase acquisition. During the acquisition, 17 lapses of >10s between triggering points were observed. The longest time lapse with no triggering was 20.4 s due to the subject falling asleep. When VBF was used, the scan time was reduced to 7.9 min (longest triggering lapse of 13.6 s, 3 lapses >10s).

**Figure 5:**
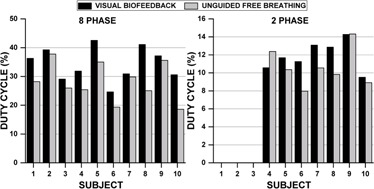
Comparison of duty cycle with and without visual biofeedback for 8‐ and 2‐phase acquisitions.

Figure 5 shows the duty cycle for all subjects. For 8‐phase acquisitions, an average duty cycle increase of 25.6%±22.7%(3.6%−64.5%) was observed with VBF. One of the largest changes was observed for Subject 8, a highly irregular breather (UFB EI−COV=17.3%, whose duty cycle increased from 25.1% without VBF to 41.1% with VBF. Applying VBF led to a decrease in total scan time of ∼4min and an improvement in absolute EI‐COV of 10.6%. By contrast, Subject 7, a regular breather (UFB EI−COV=10.3%), showed almost no improvement in duty cycle (30.9% and 29.8% with and without VBF, respectively) and negligible change in scan time (6.3 and 6.5 min with and without VBF, respectively). For the full cohort, the average duty cycle for a 2‐phase acquisition was 11.9%±1.6% for VBF and 10.6%±2.1% without.

### B. Regularity

**Figure 6:**
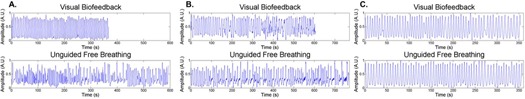
Respiratory waveform comparison of visual biofeedback (VBF) (top) and unguided free‐breathing (UFB) (bottom) for three subjects: (a) respiratory waveforms for Subject 8 showed major improvements in regularity and scan efficiency; (b) respiratory waveforms for Subject 6 displayed intermediate gains in breathing regularity and scan efficiency; (c) respiratory waveforms for Subject 9 revealed little to no improvements in breathing regularity or scan efficiency.

Table 1 summarizes the average EI‐COV, acquisition time, duty cycle, and breathing rate for both 8‐ and 2‐phase acquisitions, as well as the percent difference between VBF and UFB values for those metrics. For the 8‐phase data acquisition, a reduction in EI‐COV was observed for nine out of ten cases with VBF compared to without. The average EI‐COV reduction for the cohort was 38.8%±25.7%. Only Subject 9 showed a more regular respiratory waveform without VBF. Figure 6 shows a comparison between 8‐phase respiratory waveforms both with and without VBF for three subjects displaying (Fig. 5(a)) a high degree of irregularity (UFB EI−COV=17.3%), (Fig. 5(b)) intermediate degree of irregularity (UFB EI−COV=11.0%), and (Fig. 5(c)) a low degree of irregularity (UFB EI−COV=6.1%). Figure 6(a) provides the respiratory waveforms for Subject 8, who exhibited a highly irregular breathing pattern without VBF and displayed major improvements in breathing regularity (61.3% reduction in EI‐COV) and scan efficiency ((64.1% increase in duty cycle)) after the introduction of VBF. Figure 6(b) shows the breathing waveforms for an average subject, Subject 6. When VBF was employed, scan time was reduced by 2.7 min, a 5.3% absolute increase in duty cycle was observed, and the EI‐COV was reduced from 11.0% to 7.9%. Conversely, Subject 9 (Fig. 6(c)) showed no substantial differences after introducing VBF. The EI‐COV increased slightly with VBF compared to without (7.2% vs. 6.1%, respectively), and the absolute duty cycle difference was only 1.5%.

**Table 1:** Average end‐inhale coefficient of variation (EI‐COV), acquisition time, duty cycle and breathing rate for UFB‐VBF 8‐phase and 2‐phase acquisitions. Percent difference (%Diff) was calculated as $\frac{\text{UFB}-\text{VBF}}{\text{UFB}}\times 100\%$ for the population.

*Phases*		*EI‐COV* (Mean±SD) *(AU.)*	*Acquisition Time* (Mean±SD) *(min)*	*Duty Cycle* (Mean±SD) *(%)*	*Breathing Rate* (Mean±SD) *(breaths/min)*
Eight	VBF	8.2±4.3	7.5±2.0	34.4±5.8	11.1±3.8
	UFB	14.5±8.8	9.4±2.8	28.1±6.6	10.5±2.9
	%Diff	38.8%±25.7%	18.4%±13.2%	−25.6%±22.7%	−6.8%±29.1%
Two	VBF	7.6±4.0	4.7±1.0	11.9±1.6	10.9±2.7
	UFB	13.7±5.1	5.4±1.5	10.6±2.1	10.5±2.5
	%Diff	43.8%±16.6%	10.3%±15.8%	−14.4%±19.2	−3.8±15.0%

VBF=visual biofeedback; UFB=unguided free breathing.

For the 2‐phase acquisitions, we observed a reduction in EI‐COV of 43.8%±16.6% (range: 20.5% [Subject 10] to 61.9% [Subject 8]) with VBF, which is comparable to the 8‐phase acquisition results. For Subject 10, the improvement in regularity observed when using VBF decreased markedly between the 8‐phase and 2‐phase acquisitions (49.3% decrease in EI‐COV for 8‐phase and 20.5% decrease for 2‐phase).

### C. Qualitative artifact review

**Figure 7:**
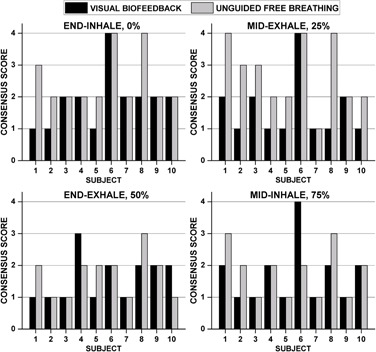
Results of qualitative artifact review performed by consensus grading (three observers) for all 10 subjects. Discontinuities in the liver dome were graded according to their severity (see text for description).

Figure 7 best summarizes the image quality consensus grading results. Differences for the EI phase (0%) neared significance (VBF=1.90±0.88,UFB=2.50±0.85,p=0.051). When all data were combined, the overall differences in image quality were statistically significant (VBF=1.70±0.85,UFB=2.18±0.93, p=0.002). Image quality was consistently better for an irregular breather (Subject 8, Fig. 6(a)) when VBF was used. On the other hand, Subject 6 showed overall poor image quality scores with no apparent improvement with the integration of VBF. Figure 6(b) illustrates that the subject's waveform was irregular even with VBF. Notably, the first few cycles of the VBF waveform that are used for the calibration had large breathing amplitudes that were not consistent with many of the other breathing cycles. In addition, both waveforms contain noise that is consistent with cardiac motion,^(23)^ which may have adversely impacted the performance of prospective triggering. Figure 2 highlights a case study where improvements in image quality were observed when VBF was used.

### D. Liver excursion

Table 2 summarizes the liver excursion along three orthogonal axes for the entire population with and without VBF. All patients showed an increase in liver excursion when VBF was employed. As expected, the liver excursion was dominant in the superior–inferior direction.

**Table 2:** Population average respiratory‐induced liver motion using centroid‐to‐centroid analysis (end‐inhale less end‐exhale).

		*Liver Excursion (mm)*		
		*S–I*	*A–P*	*L–R*
Population μ±σ(range)	VBF	14.1±4.1(8.3−20.6)	4.9±2.1(1.5−8.2)	−0.1±1.9(−3.1−2.5)
	UFB	11.9±4.5(6.8−19.5)	3.7±2.1(1.2−7.1)	0.1±1.9(−2.6−4.2)

S−I=superior−inferior; A−P=anterior−posterior; L–R=left−right; VBF=visual biofeedback; UFB=unguided free breathing; μ=mean; σ=standard deviation.

## IV. DISCUSSION & CONCLUSION

We observed a significant reduction in acquisition time and breathing irregularity after implementing VBF with 4D MRI. Using VBF led to increases in duty cycle for 8‐ and 2‐phase acquisitions. This is particularly important for an 8‐phase acquisition due to its substantially longer acquisition times where using VBF reduced scan times ∼2min. The observed liver excursion for the cohort (S‐I: 6.8–20.6 mm, A‐P: 1.2–8.2 mm, L‐R: 0–4.2 mm) agrees well with the literature (S‐I: 12–26 mm, A‐P: 1–12 mm, L–R: 1–3 mm).^(24)^ Our work revealed that incorporating VBF into 4D MRI increased liver excursion for all subjects. This suggests that VBF would need to be implemented throughout the entire radiation therapy workflow. Our findings were contrary to those obtained by Vedam et al.,^(25)^ who studied five lung cancer patients with fluoroscopy and found that diaphragm motion was comparable with and without VBF. Another study showed VBF reduced respiratory amplitude by up to 40%.^(26)^ The aim of this work was not to minimize respiratory motion but to evaluate the impact of VBF on respiratory reproducibility and scan efficiency in 4D MRI. This MR‐compatible system could also be used to guide shallow respiration and presumably reduce liver excursion.

A major complication of using prospective triggering is that changes in breathing patterns between calibration and acquisition may result in a loss of trigger efficiency, which can dramatically decrease scan efficiency.^14^, ^15^, ^16^ Using VBF mitigated this problem by producing more regular and reproducible waveforms during acquisition. Therefore, faster scan times were observed with VBF while also reducing breathing irregularities. Our results were consistent with the literature, where 40%–55% reductions in irregularity were observed when using visual and audiovisual biofeedback systems,^18^, ^19^ respectively.

Incorporating VBF into prospective 4D MRI yielded equivalent image quality ratings to UFB for half of the phase images reviewed (Fig. 7). No noticeable improvements were observed for the EE breathing phase with VBF, likely due to the longer period of time spent at end‐exhale and its known stability.^(3)^ However, 17 out of 40 paired evaluations had better image quality scores when VBF was used. The incorporation of a respiratory guiding system has shown similar improvements in image quality for 4D CT, 3D MR, and 4D PET.^27^, ^28^, ^29^ VBF tended to improve image quality for intermediate phases (i.e., mid‐exhale [i.e., 25%] and EI). This suggests that incorporating VBF may influence the overall ability to characterize the ITV at EI.

Limitations of volunteer studies include the absence of tumors and the inability to compare with 4D CT. Another limitation is that the respiratory waveform for both 4D MRI triggering and VBF was derived using two different external surrogates. While both waveforms are based on abdominal motion, ideally, the two systems would be integrated. Using external surrogates requires a confirmation of the internal/external correlation.^(3)^ Future work will involve integrating internal navigators to trigger 4D MRI acquisition, which may improve 4D MRI efficiency. Given the clear benefits in breathing regularity, image quality improvements, and image acquisition efficiency afforded by VBF, future prospective patient evaluation is warranted.

## ACKNOWLEDGMENTS

The authors would like to thank Tony Doherty at Medspira for supporting the implementation and use of the Breath Hold ES system. Technical support provided by Mo Kadbi and Tim Nielsen from Philips Medical Systems is gratefully acknowledged. Henry Ford Health System holds research agreements with Philips Medical Systems. Work partially sponsored by Henry Ford Health System Internal Mentored Grant (Carri Glide‐Hurst).

## COPYRIGHT

This work is licensed under a Creative Commons Attribution 4.0 International License.

## Supporting information

Supplementary Material

Supplementary Material
