# Supplementary figures and images for: Impact of incorporating visual biofeedback in 4D MRI
Source: J Appl Clin Med Phys. 2016 May 8;17(3):128–37. doi: 10.1120/jacmp.v17i3.6017 (PMC5690930; doi:10.1120/jacmp.v17i3.6017)

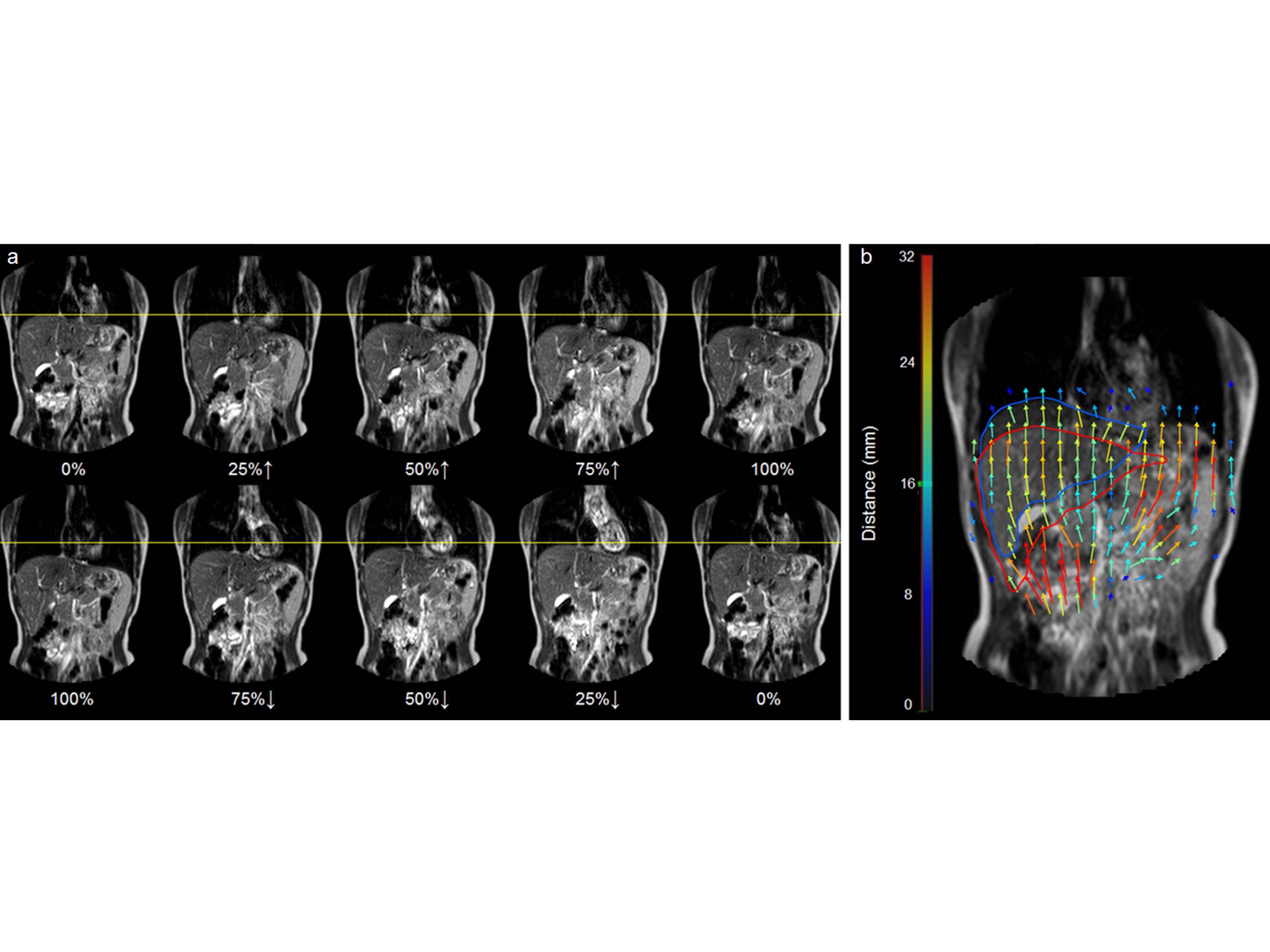

Supplement: Supplementary file 1 — Supplementary Material [file ACM2-17-128-s001.jpg]

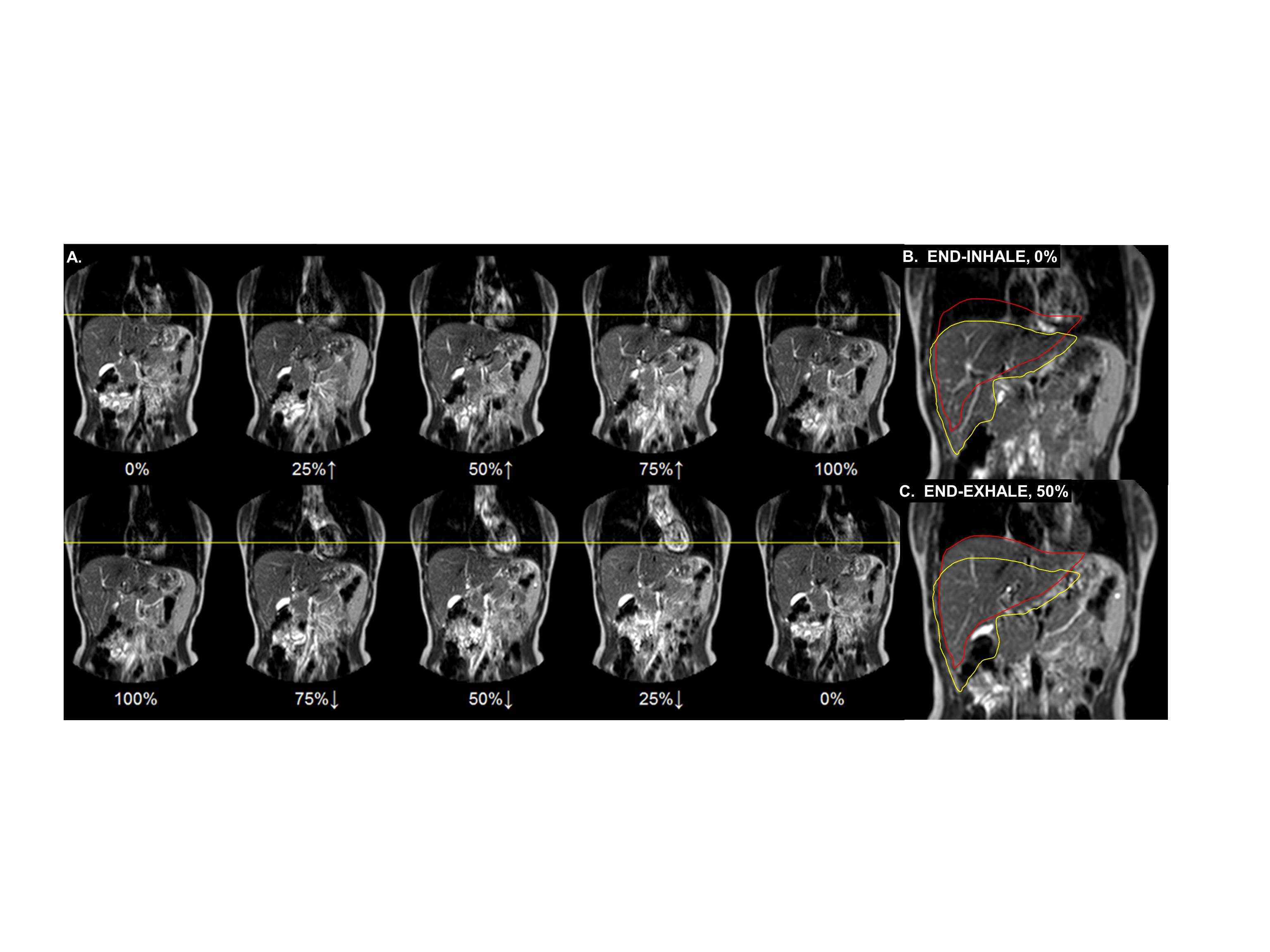

Supplement: Supplementary file 2 — Supplementary Material [file ACM2-17-128-s002.jpg]
